# Occurrence and Distribution of Carbamate Pesticides and Metalaxyl in Southern Ontario Surface Waters 2007–2010

**DOI:** 10.1007/s00128-015-1719-x

**Published:** 2016-01-11

**Authors:** John Struger, Josey Grabuski, Steve Cagampan, Ed Sverko, Chris Marvin

**Affiliations:** Water Science and Technology Directorate, Environment Canada, 867 Lakeshore Road, Burlington, ON L7S 1A1 Canada

**Keywords:** Carbamates pesticides, Carbaryl, Metalaxyl, Fungicides, Watersheds

## Abstract

Surface water sampling in 2007–2010 measured the occurrence of carbamates and metalaxyl during base flow conditions and wet weather events in southern Ontario surface waters. Carbaryl, metalaxyl and pirimicarb were the most frequently detected compounds. In 2008 these three compounds were detected in over 50 % of the samples. Overall mean concentrations of carbaryl and metalaxyl over the course of the study (2007–2010) were 15 and 18 ng/L, respectively. Elevated concentrations of carbaryl (~100 to ~950 ng/L) appeared associated with wet weather (high flow) events, while highest concentrations of metalaxyl (~20–1330 ng/L) were correlated with base flow conditions. We attributed these observations as the result of runoff of carbaryl from the watershed during rain events, while metalaxyl contamination may have resulted primarily from spray drift.

There are more than 50 compounds classified as carbamates; these compounds are heavily used in agriculture as insecticides, fungicides, herbicides, nematicides and sprout inhibitors, biocides for industrial or other applications and in household products for control of household pests. Carbamate insecticides are derivatives of carbamic acid and vary in their spectrum of activity, mammalian toxicity and persistence and are used as either dusts or sprays. The first carbamate, carbaryl, was introduced in 1956 and more of it has been used throughout the world than all other carbamates combined (Fishel 2011). Carbaryl’s low mammalian oral and dermal toxicity and broad control spectrum has resulted in wide use in lawn and garden settings (Fishel 2011).

Carbamate pesticides are derived from carbamic acid and kill insects in a similar fashion as organophosphate insecticides (Queensland Government 2002). They are effective insecticides by virtue of their ability to inhibit acetylcholinesterase in the nervous system. They can also inhibit other esterases. However, unlike the organophosphate compounds, the inhibitory effect on cholinesterase is brief. Some carbamates are translocated within plants, making them an effective systemic treatment (Queensland Government 2002).

In general, the vapour pressure of carbamates is low, although they may slowly evaporate or sublimate leading to volatilization from water and soil (IPCS 1986). Air is only considered a minor vector for transport. Aqueous systems are an important route of transport for these highly-soluble compounds. Light absorption by carbamates contributes to their rapid decomposition by photodegradation or photodecomposition under aqueous conditions. Several factors influence the biodegradation of carbamates in soil, such as volatility, soil type, soil moisture, adsorption, pH, temperature, and photodecomposition. Breakdown mechanisms affect not only the parent compound, but also products or metabolites (IPCS 1986).

Carbamate pesticides continue to be important chemicals in the Province of Ontario. Carbaryl is widely used and is currently the second-most heavily used agricultural insecticide (McGee 2010). Metalaxyl, a fungicide of the benzenoid chemical class, is used to control diseases caused by air- and soil-borne *Peronosporales* on a wide range of crops, was included in the analytical method. While technical registration of both carbofuran and pirimicarb in Canada has expired, both products were used during some of the monitoring period (2007–2010), and as of the end of 2013 were still on the residue definitions list under the Canadian Pest Control Products Act and monitored by Provincial and Federal agencies (Health Canada 2013).

Environment Canada undertook surface water sampling for carbamates and metalaxyl over the period 2007–2010 in Ontario, Canada. The purpose of this study was to measure the occurrence and distribution of carbamate pesticides and metalaxyl in southern Ontario surface waters as part of a comprehensive pesticide monitoring programme, and to compare concentrations during both base flow and wet weather events from watersheds representing a wide variety of agricultural and non-agricultural land uses.

## Materials and Methods

Water samples were collected in 1L amber glass bottles with PTFE lids affixed to a fiberglass sampling pole. Bottles and lids were rinsed twice in sample water prior to collection. Whole water samples were collected by submersing the sample bottle at mid-stream where possible to a depth of 10–20 cm below the surface and allowing to fill completely. Samples were generally collected bi-weekly through the growing season (May–September) during base flow conditions, but some peak flow (wet weather) sampling was conducted during the study beginning in 2007 and ending in 2010.

**Fig. 1:**
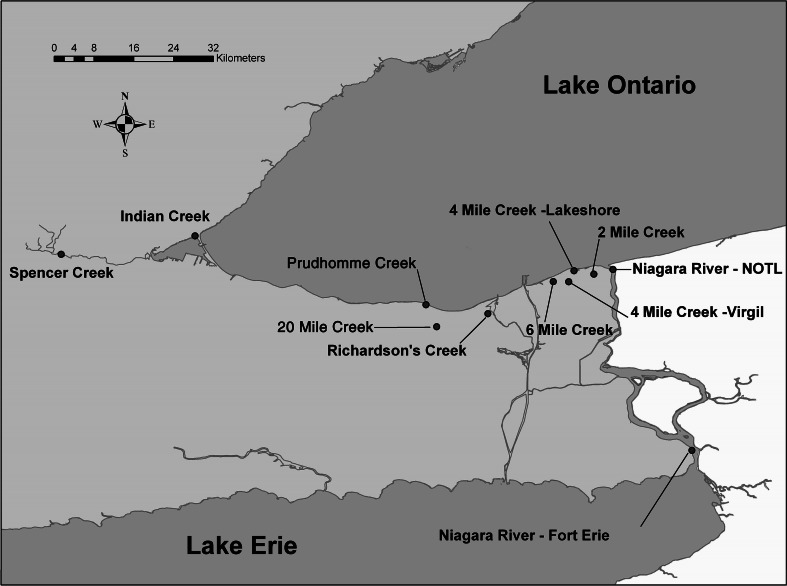
Locations of the sampling sites in southern Ontario (*NOTL* Niagara-on-the-Lake). *Note* The two sampling locations for 2 Mile Creek were represented by one location

Thirteen sites in southern Ontario consisting of nine small streams near agricultural areas, one agricultural reservoir, one urban control site (Indian Creek) that discharges an exclusively urban watershed, and two sites on the Niagara River were sampled starting in May 2007 (Fig. 1). These stream sites were selected to reflect agricultural activities including row crops, fruits and vegetables, orchards and grapes, greenhouses, ornamental nurseries and turf.

Survey design, selection of sampling locations and time of sampling over the course of the 4 year study was predicated on a number of factors, including land use, proximity to application zones, potential impacts on agricultural reservoirs, typical application periods, degree of crop rotation, and base flow conditions and peak flow conditions (wet-weather events). In this respect, the areas sampled in the Niagara Peninsula in tributary discharging areas of the southern Lake Ontario shoreline represent tremendous diversity in the types of agriculture practiced. It also included two sites on the Niagara River which flows between Lakes Erie and Ontario and is also representative of possible impacts on the Great Lakes.

Water samples (500 mL) were extracted through Supelco ENVI-Carb (6 cc, 250 mg) SPE cartridges conditioned sequentially with 10 mL 80/20 dichloromethane/methanol, 10 mL of methanol and 10 mL of deionized water. Water samples were extracted at a rate of 5 mL/min, and the SPE cartridges were dried in-line with nitrogen gas for 1 min. Cartridges were eluted with 4 mL of methanol and 8 mL of 80/20 dichloromethane/methanol at 2 mL/min, after which the extract was solvent-exchanged into methanol and concentrated to 0.9 mL for liquid chromatography–tandem mass spectrometry (LC/MS/MS) analysis.

Prior to instrument analysis, 100 μL of an internal standard containing oxamyl-d3, carbofuran-d3 and aldicarb-d3 was added to the extracts. An Agilent 1100 series high performance liquid chromatography (HPLC) system equipped with a Restek Ultra Carbamate analytical column (3.2 × 100 mm i.d., 3 μm particle size) and a mobile phase of 5 mM ammonium formate in (a) water and (b) 90 % methanol was used to separate the compounds at a flow rate of 300 μL/min in positive electrospray ionization (ESI) mode. Injection volume was 5 μL using an AB/Sciex API 2000 tandem mass spectrometer with an ESI source in positive mode.

Identification was based on one multiple reaction monitoring (MRM) transition per analyte. Analytical standards were purchased from Dr. Ehrenstorfer GmbH (Augsburg, Germany). Surface water concentrations were determined using a multi-point calibration ranging from 0.7 to 16.5 pg/μL with continuing calibration every six samples. For determination of method detection limits (MDLs), a mixed pesticide standard was spiked into deionized water and a one-sided student’s *t* distribution was used at a 99 % confidence limit. Recoveries in the spiked deionized water samples were 87 % or higher for all compounds (N = 11). Extraction efficiency of natural water samples was determined to be greater than 61 % using methomyl-d3, pirimicarb-d6 and carbaryl-d3 as surrogates. Based on results of the spiking study, instrument detection limits (IDLs) for carbamates ranged from 0.09 to 0.27 pg/μL and method detection limits (MDLs) generally ranged from 0.16 to 0.70 ng/L. Wildwood Reservoir location and data were not included in the figures because of figure scaling issues.

## Results and Discussion

**Fig. 2:**
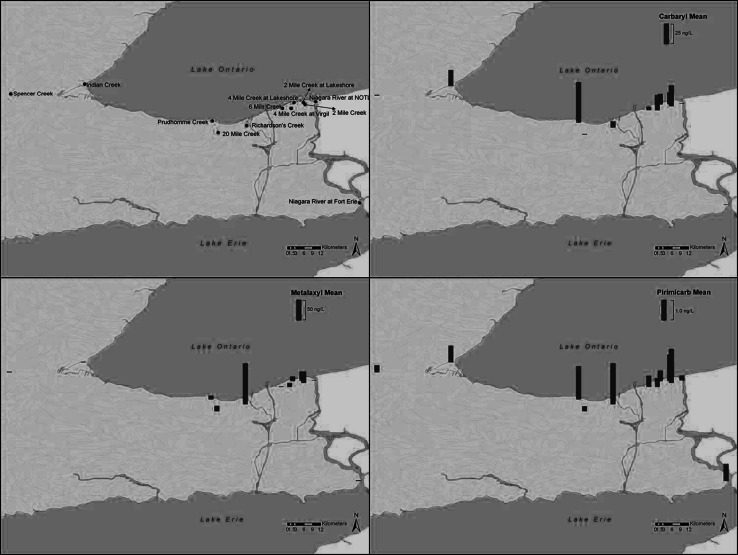
Occurrence of carbaryl, metalaxyl and pirimicarb in southern Ontario surface waters. Concentrations are expressed in ng/L

**Fig. 3:**
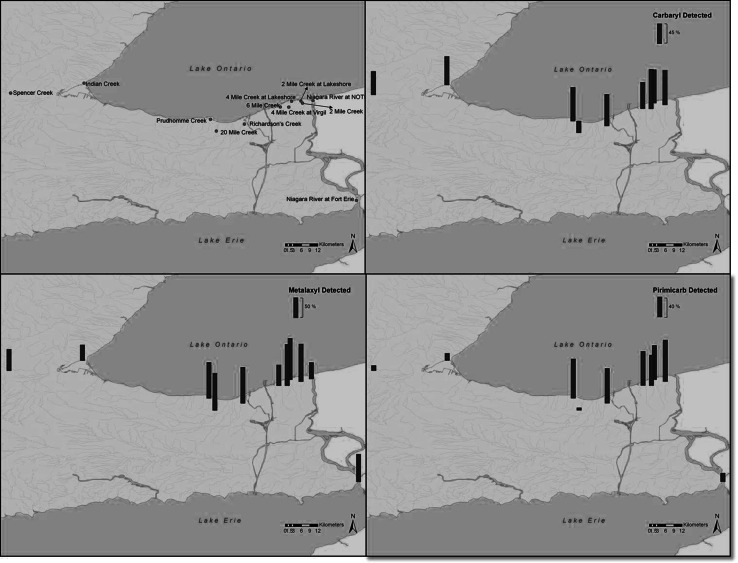
Frequency of detections for carbaryl, metalaxyl and pirimicarb in southern Ontario surface waters

Summary statistics for carbamate pesticides in individual years are shown in Table 1 while site-specific statistics are shown in Table 2. Overall summary statistics for the 2007–2010 sampling period are shown in Table 3. The occurrence of the most prevalent carbamates and their corresponding frequencies of detection are illustrated in Figs. 2 and 3, respectively.

**Table 1:** Summary statistics for concentrations (ng/L) of carbamate pesticides collected in 2007–2010 in southern Ontario

Compound	Number of samples	Number of detects	Percent detects	Mean	SD	Median	Min	Max	P25	P75	DL	GL
2007												
Aldicarb	64	0	0	–	–	–	–	–	–	–	0.70	–
Carbaryl	64	45	70	15.6	35.2	2.71	0.76	228	0.08	14.2	0.16	1
Carbofuran	64	5	7.8	–	–	–	1.37	3.26	–	–	0.24	0
Metalaxyl	64	48	75	3.04	5.91	0.95	0.42	36.5	0.26	2.87	0.42	–
Methomyl	64	0	0	–	–	–	–	–	–	–	0.30	–
Oxamyl	64	1	1.6	–	–	–	6.78	6.78	–	–	0.18	–
2008												
Aldicarb	100	0	0	–	–	–	–	–	–	–	0.70	–
Carbaryl	100	76	76	24.0	101	1.27	0.19	949	0.20	10.1	0.16	2
Carbofuran	100	20	20	–	–	–	0.26	286	–	–	0.24	0
Metalaxyl	100	88	88	18.8	37.2	2.79	0.43	176	1.01	11.1	0.42	–
Methomyl	100	23	23	–	–	–	0.46	899	–	–	0.30	–
Oxamyl	100	19	19	–	–	–	0.20	292	–	–	0.18	–
Pirimicarb	100	53	53	1.23	1.95	0.45	0.40	9.69	0.12	1.22	0.24	–
2009												
Aldicarb	46	0	0	–	–	–	–	–	–	–	0.70	–
Carbaryl	46	29	63	9.18	27.4	0.65	0.37	166	0.08	3.88	0.16	0
Carbofuran	46	13	28	–	–	–	0.25	20.2	–	–	0.24	0
Metalaxyl	46	37	80	27.4	66.8	5.89	0.48	375	1.08	20.8	0.42	–
Methomyl	46	4	8.7	–	–	–	0.41	6.41	–	–	0.30	–
Oxamyl	46	6	13	–	–	–	0.40	169	–	–	0.18	–
Primicarb	46	21	46	–	–	–	0.40	16.4	–	–	0.24	–
2010												
Aldicarb	62	0	0	–	–	–	–	–	–	–	0.70	–
Carbaryl	62	24	39	–	–	–	0.37	152	–	–	0.16	0
Carbofuran	62	1	1.6	–	–	–	2.69	2.69	–	–	0.24	0
Metalaxyl	62	31	50	27.3	169	0.42	0.63	1330	0.21	4.80	0.42	–
Methomyl	62	5	8.1	–	–	–	0.47	2.86	–	–	0.30	–
Oxamyl	62	7	11	–	–	–	1.07	14.2	–	–	0.18	–
Pirimicarb	62	12	19	–	–	–	0.24	3.63	–	–	0.24	–

Minimum value is based on the concentrations that were observed to be greater than the detection limit. Analytes reported in ng/L. GL indicates the number of detections that exceeded the CCME guidelines, while ‘–’ in the GL column indicates that no guidelines were available for these pesticides. CCME guideline for carbaryl is 200 and 1800 ng/L for carbofuran. P25 and P75 represent the 25 and 75 percentiles, respectively. DL is the detection limit in ng/L

**Table 2:** Summary statistics for concentrations (ng/L) of carbamate pesticides collected by location

Compound	Number of samples	Number of detects	Percent detects	Mean	SD	Median	Min	Max	P25	P75	DL	GL
Carbaryl											0.16	
2 Mile Creek	31	24	77	24.0	62.6	3.88	0.57	324	0.57	15.2		1
2 Mile Creek at Lakeshore	3	0	0	–	–	–	–	–	–	–		0
20 Mile Creek at Bailey	30	9	30	–	–	–	0.31	2.25	0.08	0.32		0
4 Mile Creek at Lakeshore	28	21	75	11.9	23.7	1.21	0.35	89.2	0.22	8.17		0
4 Mile Creek at Virgil	19	17	89	17.2	25.3	10.0	0.72	109	1.06	25.2		0
6 Mile Creek	20	12	60	3.33	7.22	0.61	0.57	30.7	0.08	1.50		0
Indian Creek	37	25	68	18.9	47.6	2.29	0.53	228	0.08	8.54		1
Niagara River at Fort Erie	6	0	0	–	–	–	–	–	–	–		0
Niagara River at Niagara-on-the-Lake	5	0	0	–	–	–	–	–	–	–		0
Richardson’s Creek	28	22	79	7.18	20.3	2.01	0.19	109	0.20	5.83		0
Spencer Creek at Highway #5	28	16	57	1.19	1.87	0.53	0.37	6.50	0.08	1.10		0
Prudhomme (Vineland) Creek	35	28	80	48.8	160	11.0	0.53	949	1.23	25.7		1
Wildwood Reservoir	2	0	0	–	–	–	–	–	–	–		0
Carbofuran											0.24	
2 Mile Creek	31	2	6	–	–	–	0.30	0.31	–	–		0
2 Mile Creek at Lakeshore	3	0	0	–	–	–	–	–	–	–		0
20 Mile Creek at Bailey	30	5	17	–	–	–	0.25	2.56	–	–		0
4 Mile Creek at Lakeshore	28	1	3.6	–	–	–	12.2	12.2	–	–		0
4 Mile Creek at Virgil	19	1	5.3	–	–	–	0.94	0.94	–	–		0
6 Mile Creek	20	1	5.0	–	–	–	0.41	0.41	–	–		0
Indian Creek	37	4	11	–	–	–	1.37	3.04	–	–		0
Niagara River at Fort Erie	6	0	0	–	–	–	–	–	–	–		0
Niagara River at Niagara-on-the-Lake	5	0	0	–	–	–	–	–	–	–		0
Richardson’s Creek	28	13	46	–	–	–	0.26	286	–	–		0
Spencer Creek at Highway #5	28	4	14	–	–	–	0.43	2.75	–	–		0
Wildwood Reservoir	2	0	0	–	–	–	–	–	–	–		0
Metalaxyl											0.42	
2 Mile Creek	31	28	90	27.7	58.2	4.94	0.61	251	1.23	11.7		–
2 Mile Creek at Lakeshore	3	0	0	–	–	–	–	–	–	–		–
20 Mile Creek at Bailey	30	26	87	10.9	21.1	2.56	0.92	91.0	1.45	9.44		–
4 Mile Creek at Lakeshore	28	28	100	10.7	19.7	3.46	0.42	94.1	1.47	10.1		–
4 Mile Creek at Virgil	19	19	100	9.80	23.3	2.79	0.47	98.4	0.97	4.08		–
6 Mile Creek	20	10	50	0.87	0.92	0.32	0.43	3.29	0.21	1.41		–
Indian Creek	37	15	41	–	–	–	0.45	7.01	–	–		–
Niagara River at Fort Erie	6	4	67	1.66	3.20	0.44	0.43	8.19	0.21	0.46		–
Niagara River at Niagara-on-the-Lake	5	2	40	–	–	–	0.44	0.53	–	–		–
Richardson’s Creek	28	24	86	106	251	45.6	5.85	1330	13.3	86.7		–
Spencer Creek at Highway #5	28	16	57	1.18	1.62	0.57	0.48	7.83	0.21	1.49		–
Prudhomme (Vineland) Creek	35	30	86	8.18	8.98	5.40	0.59	37.4	1.63	10.7		–
Wildwood Reservoir	2	2	100	1.48	0.57	1.48	1.07	1.88	1.07	1.88		–
Methomyl											0.30	
2 Mile Creek	31	3	9.7	–	–	–	1.90	4.51	–	–		–
2 Mile Creek at Lakeshore	3	0	0	–	–	–	–	–	–	–		–
20 Mile Creek at Bailey	30	1	3.3	–	–	–	2.86	2.86	–	–		–
4 Mile Creek at Lakeshore	28	5	18	–	–	–	1.12	85.5	–	–		–
4 Mile Creek at Virgil	19	4	21	–	–	–	0.46	92.2	–	–		–
6 Mile Creek	20	1	5	–	–	–	2.09	2.09	–	–		–
Indian Creek	37	0	0	–	–	–	–	–	–	–		–
Niagara River at Fort Erie	6	0	0	–	–	–	–	–	–	–		–
Niagara River at Niagara-on-the-Lake	5	0	0	–	–	–	–	–	–	–		–
Richardson’s Creek	28	18	64	88	226	1.00	0.41	899	0.15	11.1		–
Spencer Creek at Highway #5	28	0	0	–	–	–	–	–	–	–		–
Prudhomme (Vineland) Creek	35	0	0	–	–	–	–	–	–	–		–
Wildwood Reservoir	2	0	0	–	–	–	–	–	–	–		–
Oxamyl											0.18	
2 Mile Creek	31	4	13	–	–	–	4.06	292	–	–		–
2 Mile Creek at Lakeshore	3	0	0	–	–	–	–	–	–	–		–
20 Mile Creek at Bailey	30	0	0	–	–	–	–	–	–	–		–
4 Mile Creek at Lakeshore	28	3	11	–	–	–	0.20	0.80	–	–		–
4 Mile Creek at Virgil	19	1	5.3	–	–	–	1.98	1.98	–	–		–
6 Mile Creek	20	0	0	–	–	–	–	–	–	–		–
Indian Creek	37	2	5.4	–	–	–	0.58	1.07	–	–		–
Niagara River at Fort Erie	6	0	0	–	–	–	–	–	–	–		–
Niagara River at Niagara-on-the-Lake	5	0	0	–	–	–	–	–	–	–		–
Richardson’s Creek	28	20	71	10.2	32	0.81	0.25	169	0.09	4.30		–
Spencer Creek at Highway #5	28	0	0	–	–	–	–	–	–	–		–
Prudhomme (Vineland) Creek	35	3	8.6	–	–	–	0.35	2.41	–	–		–
Wildwood Reservoir	2	0	0	–	–	.	–	–	–	–		–
Primicarb											0.24	
2 Mile Creek	20	16	80	1.79	1.38	1.74	0.55	4.18	0.59	2.88		–
2 Mile Creek at Lakeshore	3	0	0	–	–	–	–	–	–	–		–
20 Mile Creek at Bailey	28	1	3.6	–	–	–	0.40	0.40	–	–		–
4 Mile Creek at Lakeshore	15	10	67	0.49	0.34	0.50	0.47	1.33	0.12	0.66		–
4 Mile Creek at Virgil	10	6	60	0.41	0.29	0.41	0.40	0.95	0.12	0.67		–
6 Mile Creek	9	6	67	0.52	0.40	0.46	0.44	1.20	0.12	0.67		–
Indian Creek	26	4	15	–	–	–	0.46	16.4	–	–		–
Niagara River at Fort Erie	6	1	17	–	–	–	3.97	3.97	–	–		–
Niagara River at Niagara-on-the-Lake	5	0	0	–	–	–	–	–	–	–		–
Richardson’s Creek	28	18	64	2.20	2.46	1.20	0.79	7.61	0.12	3.98		–
Spencer Creek at Highway #5	28	3	11	–	–	–	0.24	3.71	–	–		–
Prudhomme (Vineland) Creek	28	21	75	1.79	2.16	1.10	0.24	9.69	0.18	2.21		–
Wildwood Reservoir	2	0	0	–	–	–	–	–	–	–		–

Minimum value is based on the concentrations that were observed to be greater than the detection limit. Analytes reported in ng/L. GL indicates the number of detections that exceeded the CCME guidelines, while ‘–’ in the GL column indicates that no guidelines were available for these pesticides. CCME guideline for carbaryl is 200 and 1800 ng/L for carbofuran. P25 and P75 represent the 25 and 75 percentiles, respectively. DL is the detection limit in ng/L

**Table 3:** Summary statistics for concentrations (ng/L) of carbamate pesticides collected in southern Ontario from 2007 to 2010

Compound	Number of samples	Number of detects	Percent detects	Mean	SD	Median	Min	Max	P25	P75	DL	GL
Aldicarb	272	0	0	–	–	–	–	–	–	–	0.70	–
Carbaryl	272	174	64	15.2	65.6	1.01	0.19	949	0.08	8.78	0.16	3
Carbofuran	272	39	14	–	–	–	0.25	286	–	–	0.24	0
Metalaxyl	272	204	75	18.5	88.1	1.73	0.42	1330	0.32	8.14	0.42	–
Methomyl	272	32	11	–	–	–	0.41	899	–	–	0.30	–
Oxamyl	272	33	12	–	–	–	0.20	292	–	–	0.18	–
Pirimicarb	208	86	41	–	–	–	0.24	16.4	–	–	0.24	–

Minimum value is based on the concentrations that were observed to be greater than the detection limit. Analytes reported in ng/L. GL indicates the number of detections that exceeded the CCME guidelines, while ‘–’ in the GL column indicates that no guidelines were available for these pesticides. CCME guideline for Carbaryl is 200 and 1800 ng/L for Carbofuran. P25 and P75 represent the 25 and 75 percentiles, respectively. DL is the detection limit in ng/L

Aldicarb, although withdrawn from the Canadian market in 1996, was included as an analyte in the study to investigate the possibility of legacy sources or current illegal application. Used under the trademark Temik 10G, aldicarb was an insecticide widely used in agriculture in Canada, but can still be detected in water samples, especially groundwater (CCME 2009). Although the United States Environmental Protection Agency (USEPA) has implemented a ban on distribution of aldicarb by 2017, as recently as 2011 it was still used extensively in orchard and grape applications in the United States (USGS 2014). In a total of 272 samples over the course of the current study, aldicarb was not detected.

In 2010, carbofuran was targeted for phase out by 2012 by Health Canada’s Pest Management Regulatory Agency (PMRA); however, timelines have not yet been confirmed but would be determined according to normal practice (Health Canada 2010). The most recent usage data (Table 4) estimated just over 650 kg of carbofuran was used as an insecticide on crops in Ontario in 2008 (McGee 2010). Both the 2003 and 2008 reports excluded some types of agricultural use, such as greenhouse spraying, seed treatments and livestock sprays; therefore temporal comparisons between 2003 and 2008 usage data are subject to uncertainty. In the 2003 survey, total use of carbofuran was estimated at 1778 kg (McGee et al. 2004). Overall, carbofuran was detected in roughly 15 % of the total samples over the course of the study (39 of 272 samples; 14.3 %). In the last year of sampling for the study (2010), carbofuran was detected in only one sample. The United States Geological Survey (USGS) has not reported usage data for carbofuran in the United States since 2009. Of the 39 samples in which carbofuran was detected over the course of this study, one-third (13) were in Richardson’s Creek, which discharges a watershed characterized by greenhouse operations, nurseries and orchards. The Canadian Water Quality Guideline for carbofuran for the protection of aquatic life (1800 ng/L) was never exceeded in the study.

**Table 4:** Carbamate usage in the Province of Ontario (McGee et al. 2004, McGee 2010)

Compound	Usage (kg, 2003)	Usage (kg, 2008)
Pirimicarb	640	NR
Oxamyl	NR	NR
Carbaryl	4850	15,380
Carbofuran	1780	650
Metalaxyl	10,890	NR
Methomyl	560	1630

NR denotes “not reported”. Greenhouse applications not included in 2003 or 2008 data

Carbaryl has been in production in excess of 50 years and global usage since that time exceeds that of all other carbamates combined. Over the course of the survey, it was among the most frequently detected compounds, being detected in roughly 64 % of the samples (Table 3). The broad spectrum use of carbaryl was reflected in its frequent detection across the entire geographical range of sampling locations. In the United States, the primary application of carbaryl continues to be orchards and grapes (USGS 2014). Carbaryl was also detected in Indian Creek, which was the urban control stream. There was a decline in carbaryl seen in 2009 and 2010 that was likely in response to an Ontario-wide provincial ban on the sale and use of pesticides for cosmetic (non-essential) purposes (Todd and Struger 2014). Carbaryl exceeded the Canadian Water Quality Guideline for the protection of aquatic life (200 ng/L) in 3 of 272 samples; these occurred at 2 Mile Creek, Prudhomme (Vineland) Creek and Indian Creek.

Occurrences and distributions of oxamyl and methomyl were similar, presumably due to similarities in their applications. Both are used as insecticides and nematocides on row crops, ornamentals and fruits and vegetables. In the United States, these compounds are used primarily on fruits and vegetables, with some use of methomyl on corn (USGS 2014). Both oxamyl and methomyl were detected at roughly 10 %–20 % frequency at the 2 Mile Creek and 4 Mile Creek sites (Table 2). The highest frequency of detections over the course of study was at Richardson’s Creek (64 and 71 % detection frequencies for methomyl and oxamyl, respectively), with its high density of greenhouse operations. These data indicate both compounds could have found application for control of insects and nematodes on ornamentals. Limited usage data for Ontario is available for both compounds (Table 4); these data exclude greenhouse applications.

Interestingly, there has been a significant shift in application and corresponding reduction in usage of metalaxyl in the United States from primarily orchards and grapes, cotton and vegetables and fruit in the 1990s to primarily soybeans over the period 2008–2011 (USGS 2014). Data from individual years in the current study show metalaxyl was detected in at least 50 % of the samples; overall, this compound was detected at a frequency of 75 % (204 of 272 total samples) over the course of the study (Table 3). Mean annual concentrations of metalaxyl for the period of 2007–2010 showed a trend toward increasing concentrations for the first 3 years (3.04, 18.8 and 27.4 ng/L for 2007, 2008 and 2009, respectively). Metalaxyl is used as a fungicide for a wide range of applications, including row crops, vines, vegetables, ornamentals and turf. In this respect, its usage reflects the entire range of agricultural land use. Turf applications may be reflected in detection in over 40 % of the samples from Indian Creek (Table 2), which is representative of a typical urban environment. The frequency of detection in Indian Creek was second only to carbaryl (68 %, Table 2). Metalaxyl was also detected in the Niagara River at both Fort Erie and Niagara-on-the-Lake, suggesting measurable inputs and loadings into the Great Lakes.

Pirimicarb is used for control of aphids on vegetable, cereal and orchard crops. Detections were prevalent in mixed-use agricultural watersheds; overall, pirimicarb was detected in 86 of 208 total samples (41 %, Table 3). The 208 samples represent 3 years of data, compared to 4 years of data for the other compounds, as pirimicarb was not added to the analytical suite until the completion of the first year of study. No recent usage data for pirimicarb is available. The USGS reports very low usage of pirimicarb in the United States, primarily on pasture and hay (USGS 2014). Pirimicarb was also detected in the Niagara River and along with metalaxyl were the only two compounds detected in the Great Lakes connecting channel.

The usage statistics, frequency of detection and ranges of concentrations measured for carbamates were similar to those we observed for other classes of pesticides in southern Ontario, including the sulfonyl urea herbicides (Struger et al. 2011). There is a lack of applicable Canadian guidelines for the pesticide suite covered in this study; a comparison of maximum concentrations against USEPA aquatic life benchmarks resulted in no exceedances of acute benchmarks for invertebrates, and only one exceedance (899 ng/L for methomyl in 2008) of the chronic benchmark for invertebrates (USEPA 2015).

There were also some interesting differences in trends in concentrations of some compounds with respect to flow conditions. In the case of carbaryl, the highest concentrations observed in 2007, 2008 and 2010 occurred during high flow conditions that indicated watershed runoff to be the primary vector of entry into the watercourses. These observations could be rationalized given the high usage of carbaryl and its extremely broad range of applications. Conversely, over the same time period the highest concentrations of metalaxyl were correlated with base flow conditions. We attribute these observations to the potential for drift and/or overspray inputs directly to watercourses during application. The results of this study show that carbamate pesticides and metalaxyl continue to be important components of integrated pest management strategies in southern Ontario. While metalaxyl was the most-frequently detected compound, carbaryl was also detected at a rate that indicates a broad range of use five decades after its introduction. Despite its ubiquity, maximum concentrations of carbaryl rarely exceeded the CCME guideline value. The scope, frequency and temporal nature of the information presented in this study provide regulators with an understanding of the occurrence and distribution of select pesticides in surface waters, which in turn has ramifications for registration and subsequent use.
